# Risk prediction in heart failure using invasive hemodynamics

**DOI:** 10.1007/s00392-025-02690-9

**Published:** 2025-07-10

**Authors:** Martin Joachim Kraus, Aleksandre Veshapeli, Christoph Reich, Hauke Hund, Sonja Hamed, Philip W. Raake, Michael M. Kreusser, Norbert Frey, Lorenz Lehmann

**Affiliations:** 1 Department of Internal Medicine III, Division of Cardiology, University of Heidelberg, Im Neuenheimer Feld 410, 69120 Heidelberg, Germany; 2 DZHK (German Center for Cardiovascular Research), Partner Site Heidelberg/Mannheim, Mannheim, Germany; 3 Department of Cardiology, University Hospital of Augsburg, Augsburg, Germany

**Keywords:** Heart failure, Diagnostics in heart failure, Invasive hemodynamics, Risk models in heart failure

## Abstract

**Aims:**

Risk stratification in patients with heart failure patients is crucial. The prognostic value of invasive hemodynamic parameters measured by right heart catheterization compared to established risk scores remains unknown.

**Methods and results:**

This retrospective analysis included 883 patients. The combined endpoint was all-cause mortality, heart transplantation or left ventricular assist device implantation. A Cox proportional hazards model assessed the impact of invasive parameters, cardiac biomarkers, and patient characteristics, comparing them with the Seattle Heart Failure Model (SHFM) and the Meta-Analysis Global Group in Chronic Heart Failure (MAGGIC) Score. A new score was created including mean pulmonary arterial (PA) pressure, mean right atrial pressure, mean pulmonary artery wedge pressure (PAWP), age, N-terminal pro-brain natriuretic peptide (NT-proBNP), high-sensitivity troponin T (hsTnT), mixed venous oxygen saturation (SVO2), creatinine, and presence of ischemic cardiomyopathy.

Mean, diastolic, and systolic PA pressure, mean right atrial pressure, mean PAWP, SVO2 and cardiac index were significant predictors for the primary endpoint reached by 467/883 (53%) patients, in a multiple Cox proportional hazards model (*p* < 0.001). The predictive value was diminished in a subgroup of patients with ischemic cardiomyopathy. We used invasive parameters, age, NT-proBNP, hsTnT_,_ creatinine presence of ischemic cardiomyopathy and sex to develop a new model for risk stratification. This new score showed better performance compared to the SHFM and MAGGIC score in predicting the primary endpoint at 6, 12 and 24 months (area under the curve 0.76, 0.78 and 0.77 vs 0.71/0.69, 0.70/0.68 and 0.70/0.70).

**Conclusion:**

Invasive hemodynamics provides valuable measurements for predicting outcome in heart failure with reduced ejection fraction and show better performance than established risk models when combined with cardiac biomarkers and other clinical variables in this particular cohort.

**Graphical Abstract:**

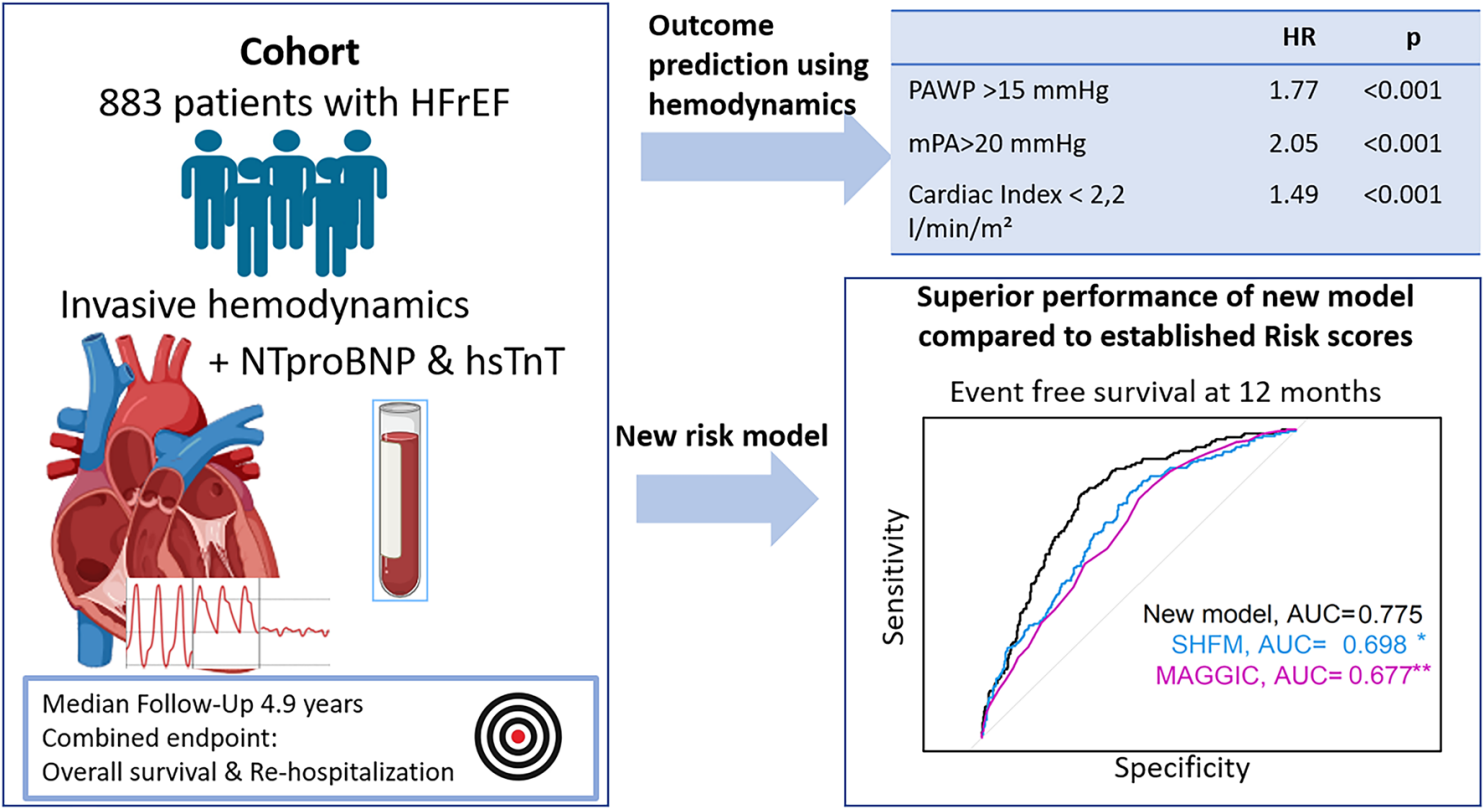

**Supplementary Information:**

The online version contains supplementary material available at 10.1007/s00392-025-02690-9.

## Introduction

Invasive hemodynamics measured by right heart catheterization represent an important tool for risk stratification in patients with heart failure. It provides an accurate measurement and estimation of important cardiac parameters, such as the cardiac index (CI), mixed venous oxygen saturation (SVO_2_), pulmonary artery wedge pressure (PAWP), pulmonary arterial pressure (PAP), pulmonary vascular resistance (PVR) as well as the assessment of right ventricular function. It is, therefore, recommended to use invasive hemodynamics as a tool for the evaluation of potential mechanical circulatory support or heart transplantation, particularly in patients with advanced heart failure [1, 2]. Several studies investigating the prognostic implications of parameters estimated by pulmonary artery catheterization have demonstrated the prognostic value of PAWP and RAP in chronic heart failure patients with reduced ejection fraction (HFrEF) and PAWP in heart failure patients with preserved ejection fraction (HFpEF) [3–6]. Furthermore, the cardiac power output or cardiac power index, as the product of CI and mean arterial pressure have been shown to be a reliable tool to predict outcome in advanced heart failure patients [7]. Cardiac Index, SVO_2_ and PAWP are part of the high urgency criteria for heart transplantation in the Eurotransplant area [8]. Risk assessment in advanced heart failure plays a very important role in the selection of possible candidates for mechanical circulatory support and heart transplantation and risk models play an important role in this process [2]. Numerous well-established risk scores are available to predict outcome in heart failure. Widely used models such as the Seattle Heart Failure Model (SHFM), the Meta-Analysis Global Group in Chronic Heart Failure (MAGGIC) Score or the Interagency Registry for Mechanically Assisted Circulatory Support (INTERMACS) staging system have been validated as excellent tools to predict outcome using clinical parameters as well as cardiac biomarkers and echocardiographic variables [9–11]. However, the prognostic value of invasive hemodynamics compared to established risk scores remains unknown. Using invasive hemodynamic parameters as part of a risk model could potentially improve survival prediction in advanced heart failure. To date, no established staging system includes invasive hemodynamics. The aim of this study was to create a useful multivariable model using clinical parameters, cardiac biomarkers and invasive hemodynamics to predict survival in heart failure and to compare the results with established heart failure models.

## Methods

The study conforms to the principles outlined in the Declaration of Helsinki [12]. The study protocol was approved by the local ethics committee. The study was conducted in a retrospective approach.

### Patient population

From January 2010 to December 2017, 2205 patients with heart failure with reduced ejection fraction underwent right heart catheterization at the department for cardiology at the University of Heidelberg (the cause for catheterization is listed in Table 1). Heart failure with reduced ejection fraction was defined as a left ventricular ejection fraction (LVEF) below 40% measured by echocardiography or as evaluated by left ventriculography. For 883 patients, high sensitivity Troponin T (hs-cTnT) as well as N-terminal pro-brain natriuretic peptide (NT-proBNP) prior to right heart catheterization were available (Supplementary Table 1). Both ambulatory and hospitalized patients were included. The patients on inotropes (*n* = 59) or intravenous diuresis were not excluded (*n* = 135). To calculate a more accurate staging system using cardiac biomarkers, patients without measurement of hs-cTnT and NT-proBNP were excluded from this analysis. Ischemic cardiomyopathy was defined as an impairment in left ventricular ejection fraction caused to a relevant part by coronary artery disease. Dilated cardiomyopathy (DCM) was diagnosed using echocardiographic parameters and cardiac magnetic resonance. If severe valve disease was the main cause of impaired left ventricular systolic function, the patients were classified as having valvular heart disease. Other causes of heart failure included cardiac amyloidosis, non-compaction-, hypertrophic-, chemotoxic-, and restrictive cardiomyopathy, hemosiderosis, myocarditis, muscular dystrophy and cardiomyopathy of unknown cause.

**Table 1:** Patient characteristics

	Overall (%)
n	883
Sex	
Female	191 (21.6)
Male	692 (78.4)
Death of any cause	333 (38.3)
Age	62 [53; 74]
Heart transplantation LVAD	72 (8.2) 63 (7.1)
Atrial fibrillation	339 (38.4%)
Permanent	64 (7.2%)
Non-permanent	275 (31.1%)
Etiology	
ICM overall Isolated ICM	359 (40.6) 164 (18.5)
ICM + DCM	30 (3.4)
ICM + valvular heart disease	151 (17.1)
DCM overall	442 (50.1)
Isolated DCM	292 (33.1)
DCM + valvular heart disease	80 (9.1)
Valvular heart disease overall	303 (34.3)
Isolated valvular heart disease	46 (5.2)
Other causes	61 (6.9)
Indication for right heart catheterization	
Valvular heart disease	217 (25.6)
Aortic valve disease	110 (50.7)
Mitral valve disease	101 (46.5)
Other	6 (2.7)
Suspected progression of heart disease	105 (11.9)
Initial workup of heart failure	233 (26.4)
Cardiac decompensation	76 (8.6)
Heart transplantation workup	222 (25%)
LVAD workup	21 (2.4%)
Emergency diagnostic	8 (0.9%)

*ICM* ischemic cardiomyopathy, *DCM* dilated cardiomyopathy, *LVAD* left ventricular assist device. Ischemic cardiomyopathy was defined as an impairment in left ventricular ejection fraction caused to a relevant part by coronary artery disease. Dilated cardiomyopathy was diagnosed using echocardiographic parameters and cardiac magnetic resonance. Values are given as median and 25% and 75% quartile or as absolute number and percent

### Patients’ workup

This included the patient’s medical history, cardiovascular risk factors, clinical assessment including evaluation of New York Heart Association (NYHA) class at the time of diagnosis. Further, complete laboratory workup including cardiac biomarkers (hs-cTnT, NT-proBNP), and serum creatinine, was done in all patients. Glomerular filtration rate (GFR) was calculated by using the Modification of Diet in Renal Disease formula. For hs-cTnT, cut-off value was < 14 pg/ml. Right heart catheterizations via a femoral venous approach were performed to determine CI, PAP, PVR and SvO_2_ as described before [13]. Cardiac index was determined by saturation measurement according to the Fick principle. The pulmonary artery pressures, mean PAWP and mean right ventricular and right atrial (RA) pressures were measured during end expiration breath hold at baseline for at least three heart cycles. Mean PAP was calculated by Metek software (Metek GmbH, Roetgen, Germany). Pulmonary vascular resistance was calculated as (mean PAP − PAWP)/cardiac output.

### Patients follow up and endpoints

For the risk stratification analysis, the combined endpoint was all-cause mortality, heart transplantation (HTX) or left ventricular assist device (LVAD) implantation. Follow-up was obtained by review of the patients’ hospital chart. If the follow-up could not be completed an inquiry to the responsible population registration was conducted. In case follow-up could not be completed, the date of the last visit was recorded as a censored event. All-cause mortality was the secondary endpoint excluding patients undergoing HTX.

### Established risk scores

Besides a new score developed in this study, previously established risk models were applied to our patient cohort. All established risk models were multivariable models for the prediction of all-cause mortality. The SHFM score was calculated by multiplying the β coefficient by the variable and summing the values as described by the authors [10]. These variables included: age, sex, NYHA class, LVEF, ischemic/non-ischemic cardiomyopathy, systolic blood pressure, use of angiotensin converting enzyme inhibitors (ACEI), use of diuretics, serum sodium concentration, hemoglobin concentration, lymphocyte count, serum uric acid concentration as well as cholesterol concentration in serum. The MAGGIC multidimensional risk score was calculated attributing points to each variable as described in the original publication [14]. Those variables included: gender, smoking status, diabetes, chronic obstructive pulmonary disease (COPD), time of heart failure diagnosis, ACEI or beta blocker use, LVEF, NYHA, creatinine concentration, body mass index (BMI), systolic blood pressure and age.

### Statistical methods

Continuous data are expressed as median and 25% and 75% percentile [Q1; Q3]. The categorical variables are expressed as absolute numbers and percentages. For the management of missing data, we employed a MICE (Multiple Imputation by Chained Equations) imputation approach to create 50 multiply imputed datasets to ensure stability of the imputations. Each missing value was imputed under a predictive model using the fully conditional specification method, where each incomplete variable is modeled conditionally given the other variables. Once imputations were complete, the analyses were performed on each of these datasets separately. The final estimates and their standard errors were combined by taking the average estimates of the 50 imputed datasets. At first variables of interest were entered in univariate Cox’ proportional hazards model with the combined endpoint as a dependent variable. In a second step invasive hemodynamic parameters were entered separately in a Cox proportional hazards model adjusted for age, sex, creatinine, presence of ICM, hs-cTnT and NT-proBNP to assess the prognostic value of invasive hemodynamics. In a next step, the multiple models were also adjusted for heart rate, atrial fibrillation during the procedure and history of atrial fibrillation. The hemodynamic parameters were not entered all together in a multiple model due to a high collinearity between the different variables. For variable selection and to test a possible benefit from using a penalized model, in a third step mean PA pressure, mean RA pressure, mean PAWP, age, NT-proBNP, hs-cTnT, SVO_2,_ creatinine and presence of ischemic cardiomyopathy were selected as predictors in a Least Absolute Shrinkage and Selection Operator (LASSO) based Cox proportional hazards model using 50 imputed datasets. As systolic and diastolic and mean PA pressure are all highly correlated variables, only mean PA pressure was chosen. Mixed venous oxygen saturation was chosen over the CI due to its superior performance in the multiple Cox’ model. As pulmonary resistance is calculated using PA pressure and PAWP it was as well excluded. The regularization parameter (lambda) in the LASSO procedure was determined through fivefold cross-validation and was chosen as the value that minimized the average cross-validated prediction error. The combined endpoint consisting of all-cause mortality, need for heart transplant or left ventricular assist device implantation was assessed as the outcome of interest. To assess whether the LASSO model has an additional value, the same variables were entered in an unpenalized Cox’ proportional hazard model. Concordance index of both models, the penalized and unpenalized was compared. We then further developed models separately for ischemic and non-ischemic cardiomyopathy. To compare the performance of the new model with established heart failure models, binary logistic regression was calculated with event free survival at 6 months, 1 year and 2 years was calculated and then compared with the SHFM and the MAGGIC scores. The comparative performance of the predictive models (new model, SHFM, and MAGGIC) was evaluated using DeLong’s test. Since the SHFM and MAGGIC Score were created to predict all-cause mortality, the comparison was also conducted with all-cause mortality as an endpoint. The results of the binary logistic regressions and DeLong’s tests were visualized using ROC curves, with the AUC providing a measure of the overall predictive accuracy of each model. Model performance was further compared using Akaike’s Information Criteria. To further investigate in which patient cohort the new model might be most useful in, it was divided into a “high risk” and “low risk” group. This was conducted using the optimal cut-off by Youden’s Index of the receiver-operator characteristics (ROC) curve predicting 1-year event free survival. The predictive value of the SHFM and MAGGIC scores was further investigated in the subcohort of patients who died within 1-year follow-up period and were classified as “high risk”. In this subgroup, the number of correctly and falsely predicted patients was counted for each score. The differences in prediction was also expressed as an absolute risk reduction to calculate a potential “number needed to catheterize”.

## Results

### Patient population

Eight-hundred-and-eighty-three patients with at least moderately reduced LVEF as defined by left ventriculography or echocardiography and complete right heart catheter workout and cardiac biomarkers including hs-cTnT and NT-proBNP were identified. Eighty-eight patients were lost to follow-up as of the 1 st of January 2020. The most frequent indication for right heart catheterization was the initial workup for heart failure (*n* = 233, (26%)) followed by catheterization as part of HTX listing (*n* = 222 (25%) and a suggested valvular heart disease (*n* = 217 (26%)). Further details about indication for invasive measurement are given in Table 1. Three-hundred-and-thirty-three patients (38%) died during follow-up, 63 patients underwent implantation of a left ventricular assist device and 72 patients received heart transplantation. The median time until the endpoint was reached was 4.9 years (IQR 1.9 years, 75% quartile not reached). Heart failure was at least partially caused by ICM in 359 (41%) cases, by DCM in 442 (50%) cases and by valvular heart disease in 303 (34%) cases. In 292 (33%) patients DCM was the only relevant cause of heart failure, 163 were diagnosed with isolated ICM (19%) and 46 (5%) with valvular heart disease as the only relevant cause of heart failure. Sixty-one cases with other causes of heart failure included 24 (3%) cases of cardiac amyloidosis, 10 (1%) cases of hypertrophic cardiomyopathy and 9 (1%) cases of cardiomyopathy with unknown cause. Other causes of heart failure were non-compaction cardiomyopathy, vasculitis, restrictive cardiomyopathy, hemosiderosis, and autoimmune related. The patient characteristics are shown in detail in Table 1 and Table 2. The missing data are listed in Supplementary Table 3.

**Table 2:** Patient characteristics

	Overall cohort (n = 883)	Ischemic Cardiomyopathy (n = 359)	Non-ischemic cardiomyopathy (n = 524)
Variable			
Use of medication			
ACEI/ARB	188 (21.7)	84 (23.7%)	104 (20.3%)
Beta blocker	628 (72.4)	265 (74.9%)	363 (70.8%)
Statins	363 (41.9)	242 (68.4%)	121 (23.6%)
MRA	404 (46.5)	162 (45.8%)	242 (47.1%)
Torasemide	437 (51.1)	165 (46,0%)	236 (46.0%)
Thiazide diuretics	54 (6.2)	36 (7.0%)	36 (7.0%)
Insulin	103 (11.7)	30 (5.8%)	30 (5.8%)
Device therapy			
CRT-D	143 (16.2)	64 (17.8%)	79 (15.1%)
Single or two chamber ICD	181 (20.5)	84 (23.4%)	97 (18.5%)
Cardiovascular risk factors			
Diabetes	251 (28.4)	153 (42.6%)	98 (18.7%)
Former or current smoker	550 (65.3)	94 (27.2%)	299 (60.2%)
Hyperlipidemia	496 (56.8)	281 (78.5%)	215 (41.7%)
Family history of myocardial ischemia	382 (43.8)	144 (40.2%)	238 (46.3%)
COPD	112 (12.8)	60 (16.8%)	52 (10.0%)
Left-bundle-branch-block	260 (31.3)	168 (39.7%)	220 (44.6%)
INTERMACS			
I	6 (0.6%)	2 (0.6%)	3 (0.6%)
II	16 (1.8)	8 (2.2%)	8 (1.5%)
III	37 (4.2)	11 (3.1%)	26 (5.0%)
IV	225 (25.5)	107 (29.9%)	118 (22.5%)
V	194 (22.0)	84 (23.5%)	110 (21.0%)
VI	111 (12.6)	42 (11.7%)	69 (13.2%)
VII	107 (12.1)	45 (12.6%)	62 (11.8%)
Less severe than VII	187 (21.2)	59 (16.5%)	128 (24.4%)
NYHA			
I	40 (4.5)	14 (3.9%)	26 (5.0%)
II	153 (17.4)	54 (15.1%)	99 (19.0%)
III	485 (55.1)	199 (55.6%)	286 (54.8%)
IV	201 (22.8)	91 (25.4%)	110 (21.1%)
Invasive hemodynamics			
Mean RA pressure (mmHg)	12.0 [8.00; 15.0]	12.0 [8.0; 15.0]	11.0 [8.0; 16.0]
Systolic PA pressure (mmHg)	45.0 [35.00; 55.0]	50.0 [40.0; 60.0]	45.0 [35.0; 55.0]
Diastolic PA pressure (mmHg)	22.5 [18.00; 28.0]	23.0 [18.5; 30.0]	22.0 [17.0; 28.0]
Mean PA pressure (mmHg)	32.0 [25.00; 40.0]	33.0 [26.0; 42.0]	31.0 [24.0; 38.0]
PVR (dyn*s/cm5)	183.0 [117; 249]	203.5 [142; 29]	63.0 [106; 229]
Mean PAWP (mmHg)	22.0 [17.0; 28.0]	22.0 [18.0; 28.0]	22.0 [16.0; 28.0]
CI (l/min/m^2^)	2.1 [1.73; 2.4]	2.0 [1.8; 2.3]	2.1 [1.7; 2.4]
SVO2 (%)	58.3 [51.3, 64.1]	56.8 [49.9; 62.0]	59.3 [52.0; 65.5]
Echocardiography			
LVEF (%)	20 [15; 30]	21 [15; 30]	20 [15; 30]
LVESD (mm)	48.0 [44.0; 52.0]	48.00 [44.0; 52.0]	48 [43; 52]
LVEDD (mm)	61.0 [55.0; 67.0]	60.00 [55.0; 66.0]	61 [55; 67]
Laboratory results			
Hemoglobin (g/l)	13.0 [11.40; 14.30]	12.2 [10.8; 13.7]	13.5 [12.0; 14.7]
NTproBNP (pg/ml)	6;237 [2575; 13337]	6;858 [3;06; 14;65]	5;741 [2;39; 12;32]
hs-cTnT (pg/ml)	33.0 [18.0; 64.0]	41.5 [25.0; 77.0]	27.0 [16.0; 56.0]
GFR (ml/min)	60.0 [41.9 79.23]	52.1 [38.6; 69.8]	63.9 [46.2; 83.1]
Creatinine (mg/dl)	1.2 [0.9; 1.5]	1.3 [1.0; 1.7]	1.1 [0.9; 1.5]
Bilirubine (mg/dl)	0.9 [0.6; 1.4]	0.90 [0.6; 1.4	1.0 [0.6; 1.4]

*ACEI* angiotensin converting enzyme inhibitor, *ARB* angiotensin receptor blocker, *MRA* mineralocorticoid receptor blocker, *CRT-D* cardiac resynchronization therapy, *ICD* implantable cardiac defibrillator, *COPD* Chronic obstructive pulmonary disease, *NYHA* New York heart association, *RA* right atrial, *PA* pulmonary artery, *PVR* pulmonary vascular resistance, *PAWP* pulmonary capillary wedge pressure, *CI* cardiac Index, *SvO2* mixed venous oxygen saturation, *LVEF* left ventricular ejection fraction, *LVEDD* Left ventricular enddiastolic diameter, *LVESD* left ventricular endsystolic diameter, *NTproBNP* N-Terminal pro-brain natriuretic peptide, *hs-cTnT* high sensitivity Troponin T, *GFR* glomerular filtration rate. Values are given as median and 25% and 75% quartile or as absolute number and percent

### Heart failure parameters

Six-hundred-and-eighty-six patients (78%) were classified as either NYHA class III or IV at baseline and 589 (67%) patients showed heart failure symptoms according to INTERMACS stage VI or lower. The hemodynamic parameters support the notion of a severe reduced LVEF with a mean CI of 2.05 [1.73; 2.40] l/min/m^2^ and mean PAWP of 22.00 mmHg [17.00; 28.00]. Mean systolic PA pressure was elevated (32.00 mmHg [25.00; 40.00]) as well as mean PVR (183 dyn*s/cm^5^ [117; 249]) [15]. Cardiac biomarkers were elevated with mean NT-proBNP of 6.237 pg/ml [2575; 13337] and mean hs-cTnT of 33 pg/ml [18; 64]. One-hundred-and-forty-three patients had cardiac resynchronization therapy (16%). All heart failure parameters are listed in Table 2.

### Cox regression

A multiple Cox’ proportional hazard model using invasive hemodynamics adjusted for age, creatinine, cardiac biomarkers, sex, and presence of ICM and the combined endpoint of all-cause mortality, heart transplantation or left ventricular assist device implantation as dependent variable revealed a statistically significant predictive value for all invasive parameters. The systolic PA pressure showed to be associated with a two percent increase in the risk of reaching the combined endpoint per unit increase in mmHg (Cox proportional hazards ratio 1.02 [1.02; 1.03], *p* ≤ 0.001). Mean RA and PA pressures showed similar results (Cox’ proportional hazard ratio 1.03 [1.02; 1.03] and 1.03 [1.02; 1.04], *p* ≤ 0.001 in both) as well as diastolic PA pressure and mean PAWP (Cox’ proportional hazard ratio 1.01 [1.00; 1.01] and 1.03 [1.01; 1.04], *p* ≤ 0.001 in both). Pulmonary arterial pressure > 20 mmHg showed a hazard ratio of 2.05 [1.38; 3.06], RA pressure > 15 mmHg a hazard ratio of 1.96 [1.57; 2.44] and PAWP > 15 mmHg was associated with a hazard ratio of 1.77 [1.29; 2.44] (*p* < 0.01 for all). Mixed venous oxygen saturation was associated with a four percent decrease in reaching the combined endpoint per % increase (Cox proportional hazards ratio 0.96 [0.95; 0.97], *p* < 0.001) and the Cardiac Index was associated with a hazard ratio of 0.67 [(0.56; 0.80], *p* < 0.001). Pulmonary vascular resistance was a significant predictor of the endpoint in the overall cohort and both subgroups (HR 1.0014, 1.002, and 1.0013 respectively, *p* < 0.001 for all). In the subgroup of patients with non-ischemic cardiomyopathy all invasive parameters showed a significant association with the combined endpoint whereas diastolic and mean PA pressure and mean PAWP were not associated in the subgroup of patients with ischemic cardiomyopathy. Results of the multiple Cox’ regression are given in detail in Table 3. Results of the univariate Cox’ proportional hazards model are listed in detail in Supplementary Table 4. We additionally calculated the model excluding patients on inotropic therapy or with diagnosis of amyloidosis to generate a more homogeneous patient cohort (Supplementary Table 6). The models were also further adjusted for heart rate and presence of atrial fibrillation during the catheter (Supplementary Table 7) and history of atrial fibrillation (Supplementary Table 8). All findings were basically replicated in the cohorts (ICMP/non-ICMP) and invasive hemodynamics remained a strong predictor for the primary outcome.

**Table 3:** Multiple Cox-regression, combined endpoint

	Overall cohort		Ischemic Cardiomyopathy		Non-ischemic cardiomyopathy	
Variable	Hazard ratio	*p*-value	Hazard ratio	*p*-value	Hazard ratio	*p*-value
Systolic PA pressure (mmHg)	1.02 [1.02; 1.03]	< 0.001	1.01 [1.00; 1.02]	0.004	1.03 [1.02; 1.04]	< 0.001
Diastolic PA pressure (mmHg)	1.01 [1.00; 1.01]	< 0.001	1.00 [1.00; 1.01]	0.246	1.03 [1.02; 1.05]	< 0.001
Mean PA pressure (mmHg)	1.03 [1.02; 1.03]	< 0.001	1.01 [1.00; 1.02]	0.089	1.03 [1.02; 1.05]	< 0.001
Mean PA pressure > 20 mmHg	2.05 [1.38; 3.06]	< 0.001	1.46 [0.46; 2.95]	0.752	2.45 [1.43; 4.20]	0.001
Mean RA pressure (mmHg)	1.03 [1.02; 1.04]	< 0.001	1.03 [1.00; 1.05]	0.040	1.03 [1.02; 1.04]	< 0.001
Mean RA pressure > 15 mmHg	1.96 [1.57; 2.44]	< 0.001	1.43 [0.72; 2.11]	0.441	2.68 [1.98; 3.63]	< 0.001
Mean PAWP (mmHg)	1.03 [1.01; 1.04]	< 0.001	1.00 [0.98; 1.02]	0.924	1.04 [1.02; 1.06]	< 0.001
Mean PAWP > 15 mmHg	1.77 [1.29; 2.44]	< 0.001	1.45 [0.53; 2.39]	0.759	2.00[1.29; 3.11]	0.002
Cardiac index (l/min/m^2^)	0.67 [0.56: 0.80]	< 0.001	1.21 [0.94; 1.56]	0.133	0.55 [0.43; 0.71]	< 0.001
Cardiac index < 2.2 l/min/m^2^	1.49 [1.20; 1.85]	< 0.001	1.06 [0.42; 1.23]	0.141	2.09 [1.52; 2.87]	< 0.001
SVO_2_ (%)	0.96 [0.95; 0.97]	< 0.001	0.99 [0.97; 1.00]	0.048	0.96 [0.94; 0.97]	< 0.001
Pulmonary vascular resistance ([dyn*s/cm^5^)	1.0014 [1.009; 1.019]	< 0.001	1.0020 [1.0010; 1.0031]	< 0.001	1.0013 [1.0006; 1.0020]	< 0.001

*PA* pulmonary artery, *RA* right atrial, *PVR* pulmonary vascular resistance, *PAWP* pulmonary capillary wedge pressure, *CI* cardiac Index, *SvO2* mixed venous oxygen saturation. Hazard ratios and p-values are the results of multiple Cox proportional hazards model. Each variable was entered separately into a model adjusted for age, creatinine, hs-cTnT, NTproBNP, sex with the combined endpoint of all-cause mortality, heart transplantation or left ventricular assist device implantation as the dependent variable. Presence of ICM was further added in the overall cohort as an additional independent variable

### LASSO Cox’ regression

Mean PA pressure, mean RA pressure, mean PAWP, age, NT-proBNP, hs-cTnT, SVO_2,_ creatinine as well presence of ischemic cardiomyopathy, age and sex were entered as independent variables into a Cox’ proportional hazards model. Following the LASSO regression, the coefficients for mean PAWP and sex were reduced to zero, indicating that these variables did not significantly contribute to the model’s predictive power in the context of other included variables. Subsequently, the same set of variables was utilized to construct a standard (unpenalized) Cox proportional hazards model. C statistic in both models regulated and unregulated showed no relevant difference in C-statistic in all imputed data sets (C-statistic 0.67 for both models). Therefore, lambda tuning with consecutive shrinkage of variables did not result in an overall improvement of event free survival prediction. We therefore decided to keep all variables as predictors for a new model.

### Comparison with established risk models

**Fig. 1:**
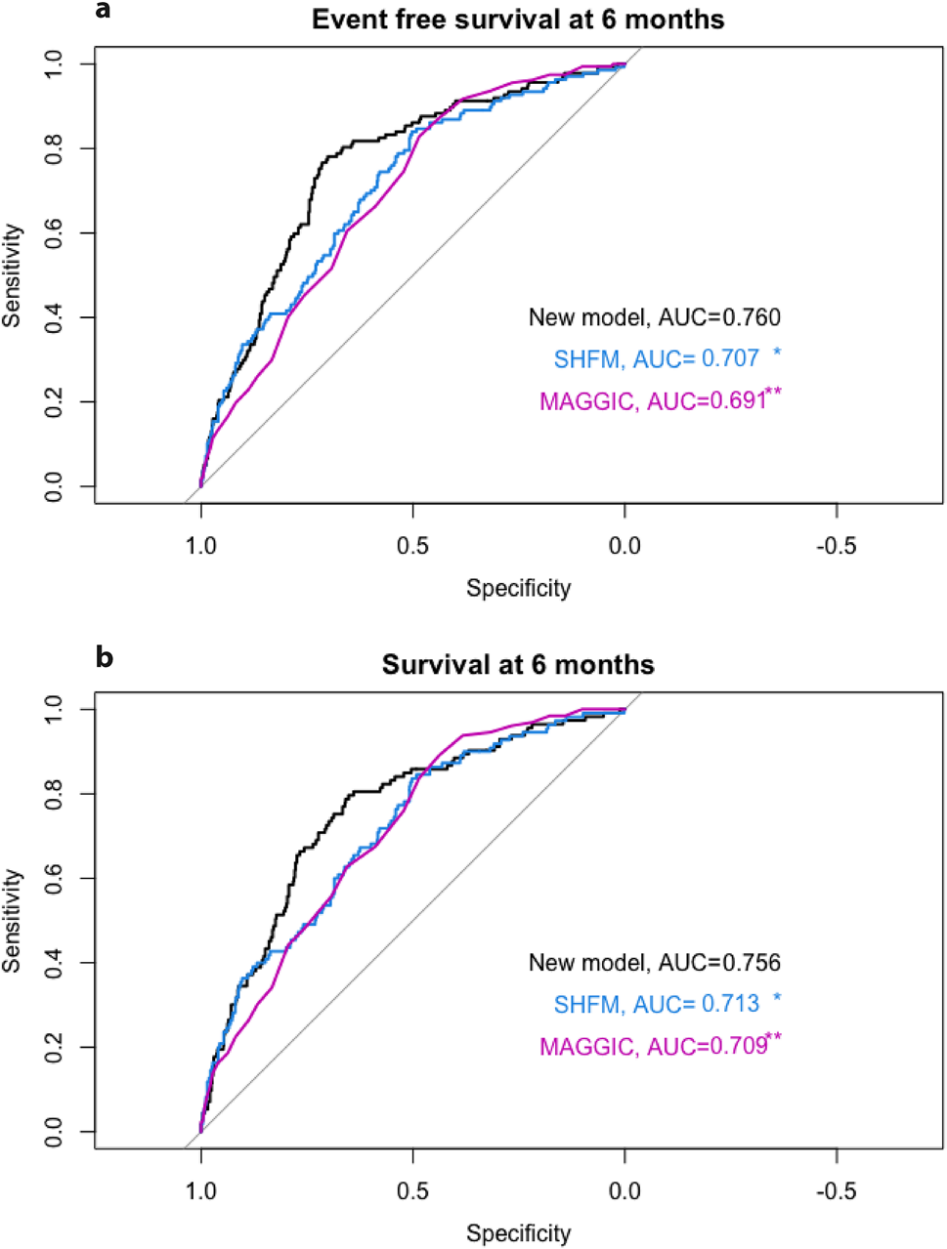
**a** Receiver operating characteristic (ROC) curve with event-free survival at six-months follow-up as a dependent variable. Each model was entered separately as an independent variable in the ROC. *AUC *area under the curve; *SHFM*   seattle heart failure model; *MAGGIC*  meta-analysis global group in chronic heart failure score. *P value of Delong test = 0.011 compared to the new model. **P-value of Delong Test = 0.001 compared to the new model. **b** Receiver operating characteristic curve with overall survival at 6 months follow-up as a dependent variable. Each model was entered separately as an independent variable in the ROC. *AUC * area under the curve; *SHFM*   seattle heart failure model; *MAGGIC*   meta-analysis global group in chronic heart failure score. *P value of Delong test = 0.043compared to the new model. **P-value of Delong Test = 0.020 compared to the new model

**Fig. 2:**
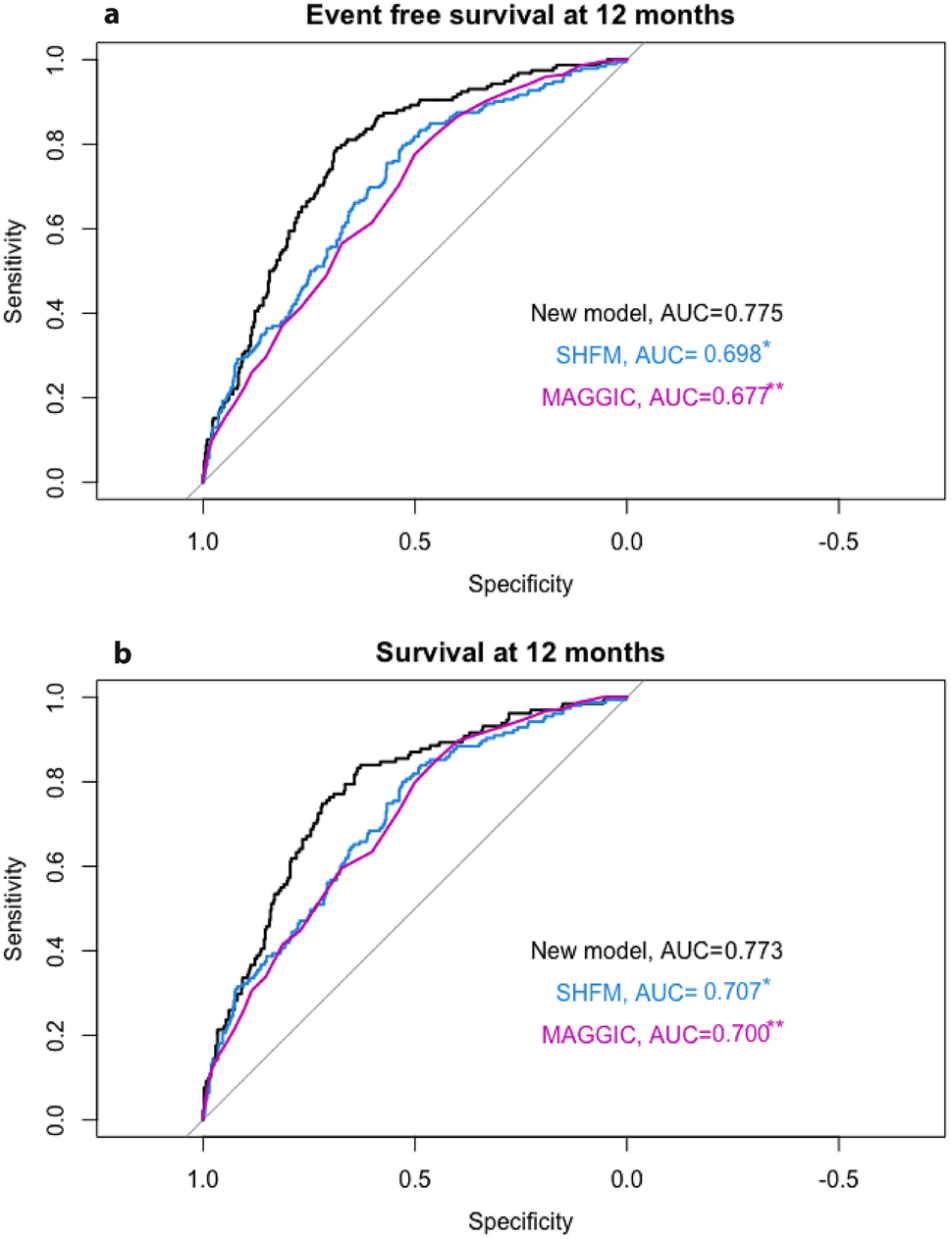
Receiver operating characteristic curve with event-free survival and overall survival at 12 months follow-up as a dependent variable. Each model was entered separately as an independent variable in the ROC. *AUC*  area under the curve; *SHFM*   seattle heart failure model; *MAGGIC*   meta-analysis global group in chronic heart failure score. *P-value of Delong Test = 0.111 compared to the new model. **P-value of Delong Test = 0.025 compared to the new model. **b** Receiver operating characteristic curve with overall survival at 12 months follow-up as a dependent variable. Each model was entered separately as an independent variable in the ROC. *AUC*  area under the curve; *SHFM*   seattle heart failure model; *MAGGIC* meta-analysis global group in chronic heart failure score. *P-value of Delong Test = 0.226 compared to the new model. **P-value of Delong Test = 0.160 compared to the new model

**Fig. 3:**
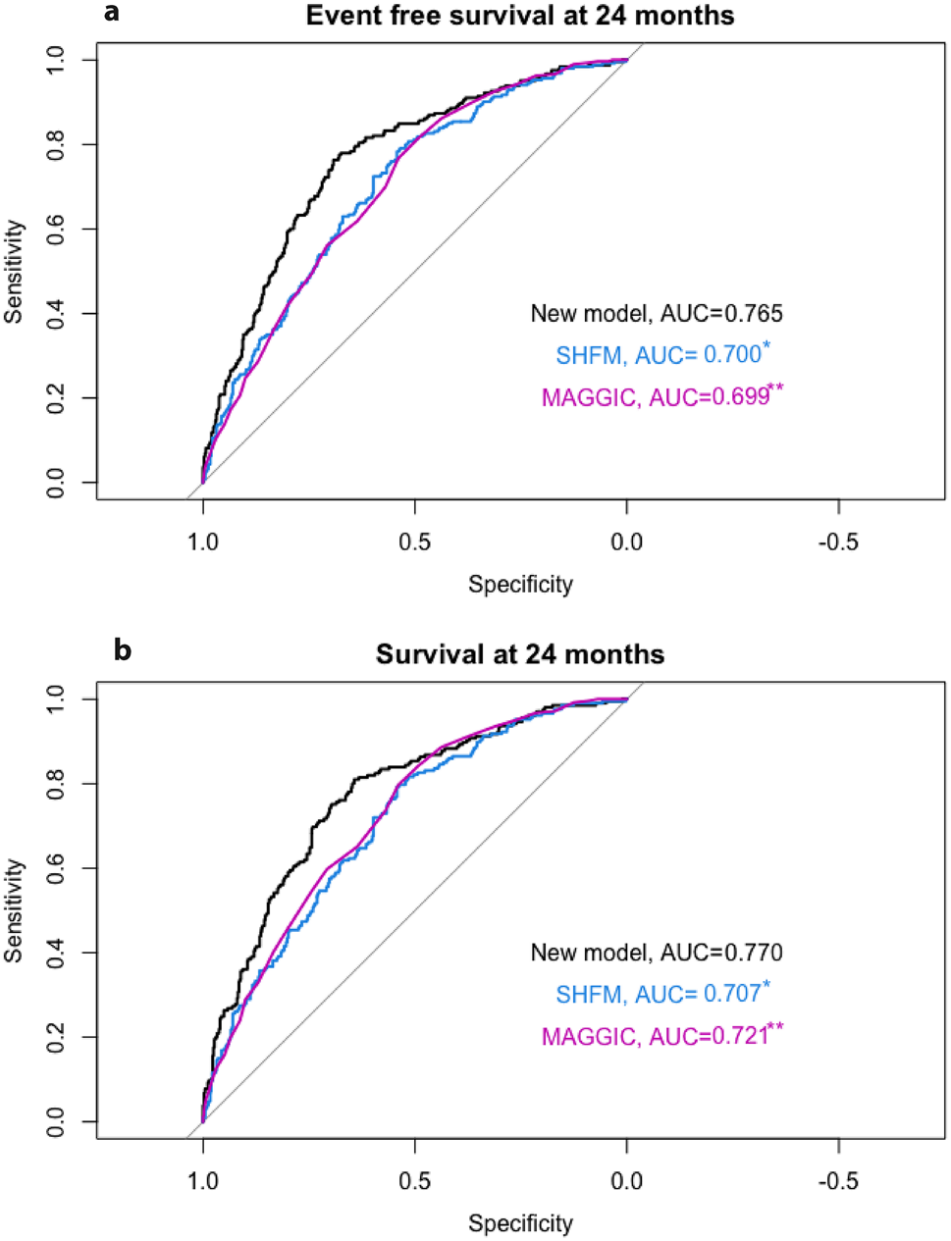
Receiver operating characteristic curve with event-free survival and overall survival at 24 months follow-up as a dependent variable. Each model was entered separately as an independent variable in the ROC. *AUC* area under the curve; *SHFM*  seattle heart failure model; *MAGGIC* meta-analysis global group in chronic heart failure score. *P-value of Delong Test = 0.020 compared to the new model. **P-value of Delong Test = 0.012 compared to the new model. **b** Receiver operating characteristic curve with overall survival at 24 months follow-up as a dependent variable. Each model was entered separately as an independent variable in the ROC. *AUC* area under the curve; *SHFM* seattle heart failure model; *MAGGIC* meta-analysis global group in chronic heart failure score. *P-value of Delong Test = 0.031 compared to the new model. **P-value of Delong Test = 0.076 compared to the new model

An overview of the different parameters used in the established and new models is given in supplementary Table 2. To compare the effect of the new model including mean PA pressure, mean RA pressure, mean PAWP, age, NT-proBNP, hs-cTnT, SVO_2,_ creatinine as well presence of ischemic cardiomyopathy, and sex with established risk scores, all three models were entered separately into a binary logistic regression model with the combined endpoint as well as all-cause mortality as a dependent variable. The newly created model showed slightly better performance for all three timepoints, 6 months, 12 months and 24 months with an area under the curve of 0.77, 0.79, and 0.77, for the combined endpoint respectively and an area under the curve of 0.77, 0.76, and 0.77 for all-cause mortality. Model performance measured by AIC was lowest in the new model for both endpoints and all timepoints except for overall survival at the six months follow-up. Akaike’s Information Criteria results are given in detail in the Supplementary Table 5. The results of the binary logistic regression are illustrated in Figs. 1a, b, 2a, b and 3a, b.

Event free survival at one year was 74% in this cohort. The SHFM predicted 92%, the MAGGIC score 73% and our model 72%. This corresponds to false prediction of outcome in 163 patients using the SHFM and 8 patients using the MAGGIC score.

**Fig. 4:**
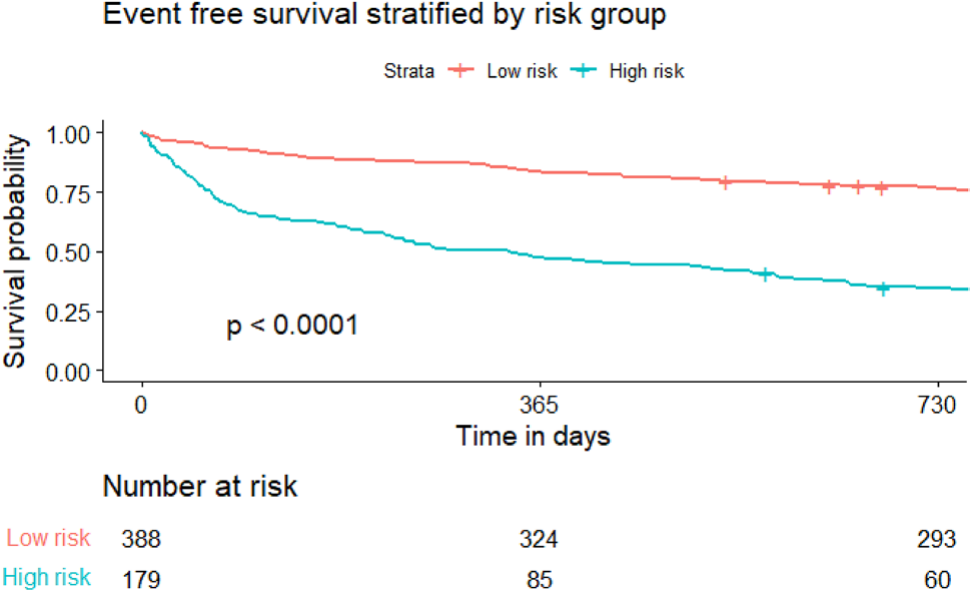
Event-free survival stratified by risk group. Risk stratification was conducted using the optimal cut-off (0.33) of the receiver-operator characteristics curve predicting event free one-year survival using a new model including mean pulmonary artery pressure, mean right atrial pressure, mean post capillary wedge pressure, age, N-terminal pro-brain natriuretic peptide, high sensitivity troponin T, mixed venous oxygen saturation, creatinine as well presence of ischemic cardiomyopathy and sex. *P*-value is the result of a log-rank test. Vertical bars indicate censored events

Using Youden’s Index for an optimal cut-off of 0.33 to predict 1-year event free mortality, the new model was divided into a “high risk” and “low risk” group. Event free survival is further illustrated in Fig. 4. One-hundred-and-forty-two patients out of 172 (82%) “high risk” patients died within 1 year. This cohort was characterized by an elevated mean NT-proBNP (12,438 pg/ml [5912; 23603]), low CI (1.77 l/min [1.51; 2.10]) and low SVO2 (48% [44; 52]) when compared to the overall cohort and rather moderately elevated hs-cTnT of 55.00 pg/ml [28.00, 89.50]. In this sub cohort, the predicted 1-year mortality using the SHFM was 13% and 30% using the MAGGIC score while the actual one-year mortality was 66%, as predicted by the new model. Therefore, in this “high-risk” cohort the SHFM would have underestimated a fatal outcome in 76 patients and the MAGGIC score in 51 patients. In the “low risk” subgroup no patients died within the first year. Predicted mortality according to SHFM was 11% and 28% using the MAGGIC Score while the new model predicted a one-year mortality of 0.

## Discussion

The present study aims to classify the value of invasive hemodynamics in predicting outcome compared to established risk stratification systems. We calculated a new scoring system using invasive hemodynamics as well as established cardiac biomarkers, laboratory, and clinical findings. This scoring system, at least when adapted to patients with heart failure with reduced ejection fraction from our centre, showed a superior performance compared to published predictive scores as the SHFM or the MAGGIC scoring system.

We were able to demonstrate that the results of invasive hemodynamic testing using right heart catheterization are reliable tools for predicting outcome in heart failure with reduced ejection fraction. We have also shown that invasive hemodynamics perform better in patients with non-ischemic cardiomyopathy than in patients with ischemic cardiomyopathy.

A first and important finding of this study is the strong predictive value of hemodynamic parameters separately. Elevated Mean PA pressure > 20 mmHg for example was associated with a hazard ratio for reaching the combined endpoint of 2.05 [1.38; 3.06] while an elevated PAWP above 15 mmHg and a Cardiac Index < 2.2 l/min/m^2^ were both also associated with a very high risk of HTX, LVAD implantation or death (HR 1.77 [1.29; 2.44] and 1.49 [1.20; 1.85], *p* < 0.001). In comparison to this the risk of reaching the endpoint increased by 0.5–1% per 1000 unit increase in NTproBNP (pg/ml*1000). These findings align with previous findings demonstrating the good predictive value of invasive hemodynamic parameters in patients with preserved ejection fraction [16]. We also demonstrated the high prognostic value of PVR which aligns with prior studies demonstrating this effect in large cohorts with preserved and reduced ejection fraction [16, 17]. In addition, we could demonstrate, as shown in a multiple Cox’ proportional hazards model for mean PA pressure, mean RA pressure, mean PAWP and SVO2 are that invasive parameters are independent predictors of outcome in patients with heart failure. It is worth noting that these variables represent values related to cardiac filling pressure and cardiac output.

A second aim of this study was to determine which group of patients benefits the most from invasive hemodynamics. In our study, all invasive parameters showed a better predictive value in the subcohort of patients with non-ischemic cardiomyopathy. This effect was particularly strong for the CI with a hazard ratio of 0.55 [0.43; 0.71]. A potential explanation could be that invasive parameters do not reflect the extent of coronary artery disease. If these findings would be consistent in further studies, they could carry larger implications since ischemic cardiomyopathy is a very common reason for heart failure and invasive hemodynamic is still a very important prognostic tool for HTX planning in this patient group [8]. Still, these findings must be treated with caution. However, in patients without significant coronary artery disease, invasive hemodynamic parameters showed to be excellent predictors of outcome. In this subgroup, they may be particularly helpful in making decisions about LVAD implantation and HTX planning.

A third part was to investigate the additive value of combining invasive parameters with cardiac biomarkers. We created a new model using mean PA pressure, mean RA pressure, mean PAWP, age, NT-proBNP, hs-cTnT, SVO_2_, creatinine as well presence of ischemic cardiomyopathy. In our patient cohort this model showed better performance than the well-established SHFM and the MAGGIC Score. The C-statistic of 0.67 of the new model was comparable to the performance of the SHFM and MAGGIC score in prior studies [10, 11, 18].

The new scoring system showed equivalent performance to other risk models in a subcohort of patients with very high NT-proBNP 12438 pg/ml [5912; 23603], low CI (1.77 l/min/m^2^ [1.51, 2.10]) and moderate hs-cTnT 55 pg/ml [28; 89]. This finding supports the usefulness of invasive parameters and cardiac biomarkers in the setting of advanced heart failure and is consistent with the usage of CI, PAWP and SVO_2_ as parameters for high urgency listing for HTX. Taken together, the use of both NTproBNP and invasive hemodynamic parameters may be very useful parameters for predicting outcome in heart failure in non-ischemic patients, potentially outperforming established risk scores.

To compare the new model with established risk scores the underlying patient populations must be considered. Our patient cohort shows a higher percentage of patients NYHA III and IV patients (77.9%) when compared to the cohorts used for the MAGGIC score (39.7%) and similar to the PRAISE1 cohort used in the SHFM (median NYHA class 3.6) [10, 11]. Median age was similar in all three groups (62, 64, and 65 years, respectively) while ACEI and beta-blocker use differed in all three cohorts (21.7% vs 68% and 0% respectively for ACEI, and 72% vs 40% vs 0% for beta blockers). Median VEF was 36.6% in the cohort used for the MAGGIC score and 21.6% in the SHFM. Considering a median CI of 2.05 l/min/m^2^ in our cohort it can be concluded that the population used in this study is comparable to the cohort used to create the SFHM in terms of heart failure parameters and represents a more advanced stage of heart failure when compared to the MAGGIC score.

However, the potential risks of right heart catheterization (e.g., bleeding risk) must be considered when discussing the benefits in predicting outcome. In this cohort, eight patients who died within 12 months would not have been identified as high risk by the MAGGIC score and 163 would have been missed by the SHFM. Taking the risk reduction of 10% and 1% into consideration this would roughly correspond to a “number needed to catheterize” of 100 and 10, respectively. In the mentioned subgroup of high-risk patients, the MAGGIC Score would have missed 51 patients out of 94 patients who died within 1 year and the SHFM 76 out of 94 patients. In this subcohort, only three patients would need right heart catheterization to improve survival prediction when compared to the MAGGIC score and only two when compared to the SHFM.

### Limitations

This study was conducted in a single-centre retrospective approach and data was not complete. There was no external validation cohort. The patients without complete NTproBNP were excluded in this study, which could contribute to a selection bias. Another limitation is the heterogenous patient population we used. Both ambulatory and hospitalized patients were included as well as patients on inotropes and at very different stages of heart failure. This is also the explanation for the big differences in medication between patients. The vastly different indications for invasive measurement are another cause of bias. However, considering the positive results for hemodynamic measurement underlines the utility of right heart catheterization in a broad patient population. Although there are limitations, such as the reliance on Fick’s principle for invasive hemodynamic measurements, the study provides a foundation for potential advancements. While acknowledging the need for further validation in a separate cohort, the newly developed model holds promise for improving our understanding of cardiac dynamics and patient outcomes. It must be stated that the SFHM and MAGGIC are universal models that can be applied to both patients with HFrEF and HFpEF and have been sufficiently validated in external cohorts. However, the main goal of these models is to predict all-cause mortality. Predicting hospitalization with these models must be done with caution. In addition, invasive measurement is a procedure with potential risks and complications which heart failure models do not carry. The fact that invasive hemodynamic parameters performed better in patients with non-ischemic cardiomyopathy could potentially be attributed to a Type II error, and therefore, by chance. However, the findings were consistent for multiple parameters adjusted for different potential confounders. Still, as mentioned above, further external validation is needed.

## Conclusion

In summary, the study involved 883 patients with heart failure, examining invasive hemodynamic parameters for predictive modeling. The developed model, incorporating factors like pulmonary artery pressure and cardiac index, demonstrated superior performance compared to established risk scores in predicting outcomes, emphasizing its potential clinical utility.

## Supplementary Information

Below is the link to the electronic supplementary material.

Supplementary file1 (DOCX 74 KB)

## Data Availability

The data that support the findings of this study are not openly available due to reasons of sensitivity and are available from the corresponding author upon reasonable request.
