# Supplementary material for: Risk prediction in heart failure using invasive hemodynamics
Source: Clin Res Cardiol. 2025 Jul 10;114(10):1388–99. doi: 10.1007/s00392-025-02690-9 (PMC12460544; doi:10.1007/s00392-025-02690-9)
Supplement: Supplementary file 1 — Supplementary file1 (DOCX 74 KB) [file 392_2025_2690_MOESM1_ESM.docx]

**Appendices**

**Supplementary Table 1 – Patient Flow-diagram**


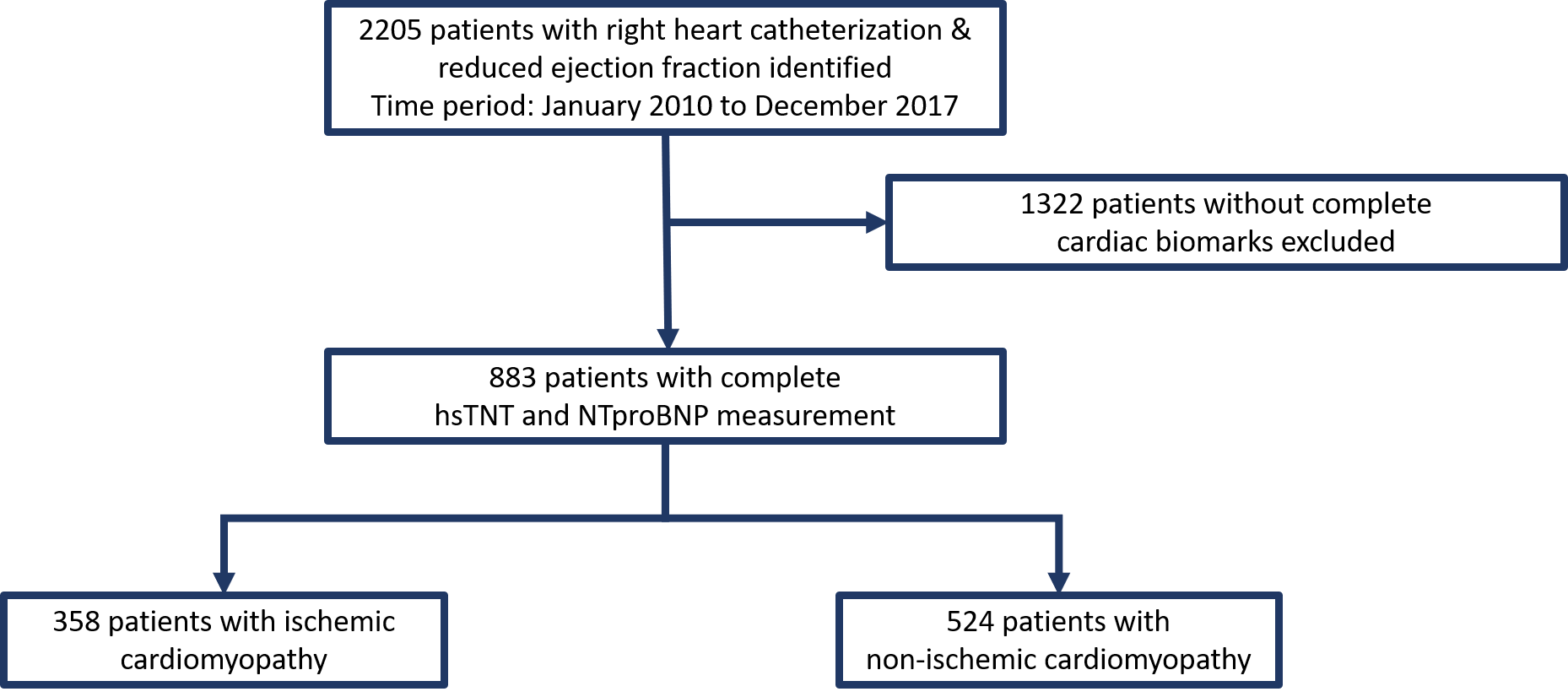
hs-cTnT = high sensitivity Troponin T, NTproBNP= N-Terminal pro-Brain Natriuretic Peptide

**Supplementary Table 2–Risk model variables**

|  |  | **SHFM** | **MAGGIC** | **New Model** |
| --- | --- | --- | --- | --- |
| **Clinical data /**  **Clinical history** | Gender | X | X | X |
|  | Age | X | X | X |
|  | Weight/BMI | X | X |  |
|  | NYHA | X | X |  |
|  | Systolic RR | X | X |  |
|  | Heart Failure medication | X | X |  |
|  | Device therapy | X |  |  |
|  | Diabetes |  | X |  |
|  | Smoker |  | X |  |
|  | COPD |  | X |  |
|  | Timepoint of diagnosis |  | X |  |
|  | ICM |  |  | X |
| **Labratory results** | Hemoglobin | X |  |  |
|  | Sodium | X |  |  |
|  | Total Cholesterol | X |  |  |
|  | Urica Acid | X |  |  |
|  | Lymphocytes | X |  |  |
|  | Creatinin |  | X | X |
|  | NtproBNP |  |  | X |
|  | hs-cTnT |  |  | X |
| **Echocardiography** | LVEF | X | X |  |
| **Hemodynamic parameters** | Mean PA pressure |  |  | X |
|  | Mean RA pressure |  |  | X |
|  | SVO_2_ |  |  | X |
|  | PAWP |  |  | X |

Overview of variables in the three different risk models. BMI = Body mass index, NYHA = New York Heart Association functional class, RR = blood pressure, COPD = Chronic obstructive pulmonary disease, ICM = Ischemic cardiomyopathy, NTproBNP = N-terminal pro-brain natriuretic peptide, hs-cTnT= high sensitivity troponin T, PA = pulmonary artery, RA = right atruial, SVO_2_ = mixed venous oxygen saturation, PAWP = post capillary wedge pressure.

**Supplementary Table 3 -Missing Data**

| **Variable** | **Number of missing values in the overall collective (n=883) (%)** |
| --- | --- |
| **Use of medication** |  |
| ACEI/ARB | 33 (2.7) |
| Beta blocker | 16 (1.8) |
| Statins | 16 (1.8) |
| MRA | 15 (1.7) |
| Torasemide | 16 (1.8) |
| Thiazide diuretics | 16 (1.8) |
| Insulin | 6 (0.7) |
| **Device therapy** |  |
| CRT-D | 3 (0.34) |
| Single or two chamber ICD | 3 (0.34) |
| **Cardiovascular risk factors** |  |
| Diabetes | 6 (0.7) |
| Former or current smoker | 79 (8.9) |
| Hyperlipidemia | 10 (1.1) |
| Family history of myocardial ischemia | 11 (1.3) |
| COPD | 6 (0.7) |
| Left-bundle-branch-block | 52 (5.9) |
| **INTERMACS** | 0 |
| **NYHA** | 4 (0.5) |
| **Invasive hemodynamics** |  |
| Mean RA pressure (mmHg) | 16 (1.81) |
| Systolic PA pressure (mmHg) | 11 (1.3) |
| Diastolic PA pressure (mmHg) | 11 (1.3) |
| Mean PA pressure (mmHg) | 11 (1.3) |
| PVR (dyn*s/cm^5^) | 25 (2.8) |
| Mean PAWP (mmHg) | 33 (3.7) |
| CI (l/min/m²) | 14 (1.6) |
| SVO_2_ (%) | 46 (5.2) |
| **Echocardiography** |  |
| LVEF (%) | 471 (53%) |
| LVEDD (mm) | 257 (29%) |
| LVESD (mm) | 257 (29%) |
| **Labaratory results** |  |
| Hemoglobin (g/l) | 0 |
| NTproBNP (pg/ml) | 0 |
| hsTnT (pg/ml) | 68 (7.7) |
| GFR (ml/min) | 0 |
| Creatinine (mg/dl) | 0 |
| Bilirubine (mg/dl) | 168 (19.0) |

ACEI= Angiotensin converting enzyme inhibitor, ARB= Angiotensin receptor blocker, MRA= Mineralocorticoid receptor blocker, CRT-D= Cardiac resynchronization therapy, ICD= implantable cardiac defibrillator, COPD =Chronic obstructive pulmonary disease, LVEF= Left venctriular ejection fraction, LVEDD = Left ventricular enddiastolic diameter, LVESD= Left ventricular endsystolic diameter, NYHA= New York Heart Association, RA= Right atrial, PA= pulmonary artery, PVR= pulmonary vascular resistance, PAWP= pulmonary capillary wedge pressure, CI= Cardiac Index, SvO2= mixed venous oxygen saturation, NTproBNP= N-Terminal pro-Brain Natriuretic Peptide, hs-cTnT = high sensitivity Troponin T, GFR= Glomerular filtration rate. Values are given as absolute number and percent. The difference in missing values of SVO2 and CI is caused by missing data and is not attributed to other types of CI measuring.

**Supplementary Table 4- Univariate Cox-Regression for the combined endpoint**

|  | **Overall cohort** | | **Ischemic Cardiomyopathy** | | **Non-ischemic cardiomyopathy** | |
| --- | --- | --- | --- | --- | --- | --- |
| **Variable** | **Hazard ratio** | **p-value** | **Hazard ratio** | **p-value** | **Hazard ratio** | **p-value** |
| **Systolic PA pressure (mmHg)** | 1.02 (1.02: 1.03) | <0.001 | 1.01 [1.00; 1.02] | 0.004 | 1.03 [1.02; 1.04] | <0.001 |
| **suDiastolic PA pressure (mmHg)** | 1.01 [1.00; 1.01] | <0.001 | 1.00 [1.00; 1.01] | 0.246 | 1.03 [1.02; 1.05] | <0.001 |
| **Mean PA pressure (mmHg)** | 1.03 (1.02; 1.03) | <0.001 | 1.01 [1.00; 1.02] | 0.089 | 1.03 [1.02; 1.05] | <0.001 |
| **Mean RA pressure (mmHg)** | 1.03 [1.02; 1.04] | <0.001 | 1.03 [1.00; 1.05] | 0.040 | 1.03 [1.02; 1.04] | <0.001 |
| **Mean PAWP (mmHg)** | 1.03 [1.01; 1.04[ | <0.001 | 1.00 [0.98; 1.02] | 0.924 | 1.04 [1.02; 1.06] | <0.001 |
| **Cardiac index (l/min/m²)** | 0.68 (0.56; 0.80) | <0.001 | 1.21 [0.94; 1.56] | 0.133 | 0.55 [0.43; 0.71] | <0.001 |
| **SVO_2_ (%)** | 0.96 [0.95; 0.97] | <0.001 | 0.99 [0.97; 1.00] | 0.048 | 0.96 [0.94; 0.97] | <0.001 |
| **Pulmonary vascular resistance (dyn*s/cm^5^)** | 1.001 [1.0009; 1002] | <0.001 | 1.002 [1.001; 1.003] | <0.001 | 1.001 [1.0006; 1.001] | <0.001 |
| **Age (years)** | 1.01[1.01; 1.02] | <0.001 | 1.02 [1.01; 1.04] | <0.001 | 1.01 [1.01; 1.02] | 0.001 |
| **NTproBNP (ng/l)** | 1.00001[1.000006; 1.00001] | <0.001 | 1.000007 [1.000004; 1.00001] | <0.001 | 1.0001 [1.00001; 1.00002] | <0.001 |
| **hsTnT (pg/ml)** | 1.0003 [1.00004, 1.0006] | 0.026 | 1.0006 [1.00009; 1.001]] | 0.020 | 1.0001 [0.99; 1.00] | 0.567 |
| **Presence of ICM** | 1.83 [1.52; 2.19] | <0.001 |  |  |  |  |

PA = pulmonary artery, RA = Right atrial, PVR = pulmonary vascular resistance, PAWP = pulmonary capillary wedge pressure, CI = Cardiac Index, SvO2 = mixed venous oxygen saturation, NTproBNP = N-Terminal pro-Brain Natriuretic Peptide, hsTNT = high sensitivity Troponin T, ICM = ischemic cardiomyopathy. Hazard ratios and p-values are the results of univariate Cox proportional hazards model with the combined endpoint of all-cause mortality, heart transplantation or left ventricular assist device implantation as the dependent variable.

**Supplementary Table 5 – Akaike’s Information Criteria**

|  | **6 months** | | **12 months** | | **24 months** | |
| --- | --- | --- | --- | --- | --- | --- |
|  | **Overall survival** | **Event free survival** | **Overall survival** | **Event free survival** | **Overall survival** | **Event free survival** |
| **New model** | 559 | 628 | 525 | 191 | 702 | 781 |
| **SHFM** | 542 | 629 | 646 | 742 | 720 | 814 |
| **MAGGIC** | 641 | 741 | 766 | 881 | 839 | 953 |

Akaikes’s Information Criteria for all three timepoints for the three different models. SHFM= Seattle Heart Failure Model; MAGGIC= Meta-Analysis Global Group in Chronic Heart Failure Score. Numbers indicate numbers of patients.

**Supplementary Table 6 -– Multiple Cox-Regression for the combined Endpoint - excluding patients with cardiac amyloidosis and patients on inotropes**

|  | **Overall cohort**  **(n=784)** | | **Ischemic Cardiomyopathy (n=320)** | | **Non-ischemic cardiomyopathy (n=464)** | |
| --- | --- | --- | --- | --- | --- | --- |
| **Variable** | **Hazard ratio** | **p-value** | **Hazard ratio** | **p-value** | **Hazard ratio** | **p-value** |
| Systolic PA pressure (mmHg) | 1.02 [1.02; 1.03] | <0.001 | 1.01 [1.00; 1.02] | 0.004 | 1.03 [1.02; 1.04] | <0.001 |
| Diastolic PA pressure (mmHg) | 1.01 [1.00; 1.01] | <0.001 | 1.00 [1.00; 1.01] | 0.243 | 1.04 [1.02; 1.05] | <0.001 |
| Mean PA pressure (mmHg) | 1.03 [1.02; 1.03] | <0.001 | 1.01 [1.00; 1.02] | 0.115 | 1.03 [1.02; 1.05] | <0.001 |
| Mean RA pressure (mmHg) | 1.03 [1.02; 1.04] | <0.001 | 1.02 [1.00; 1.05] | 0.101 | 1.03 [1.02; 1.05] | <0.001 |
| Mean PAWP (mmHg) | 1.03 [1.01; 1.04] | <0.001 | 1.00 [0.98; 1.01] | 0.760 | 1.05 [1.03; 1.06] | <0.001 |
| SVO_2_ (%) | 0.96 [0.95; 0.97] | <0.001 | 0.99 [0.97; 1.59] | 0.122 | 0.95 [0.93; 0.96] | <0.001 |
| Pulmonary vascular resistance ([dyn*s/cm^5^) | 1.0013 [1.0158; 1.019] | <0.001 | 1.0022 [1.0011; 1.0033] | <0.001 | 1.0010 [1.0002; 1.0017] | 0.011 |

PA = pulmonary artery, RA = Right atrial, PVR= pulmonary vascular resistance, PAWP = pulmonary capillary wedge pressure, SvO2= mixed venous oxygen saturation. Hazard ratios and p-values are the results of multiple Cox proportional hazards model. Each variable was entered separately into a model adjusted for age, creatinine, hs-cTnT, NTproBNP, sex with the combined endpoint of all-cause mortality, heart transplantation or left ventricular assist device implantation as the dependent variable. Presence of ICM was further added in the overall cohort as an additional independent variable.

**Supplementary Table 7 -– Multiple Cox-Regression for the combined Endpoint – adjusted for heart rate and atrial fibrillation**

|  | **Overall cohort**  **(n=784)** | | **Ischemic Cardiomyopathy (n=320)** | | **Non-ischemic cardiomyopathy (n=464)** | |
| --- | --- | --- | --- | --- | --- | --- |
| **Variable** | **Hazard ratio** | **p-value** | **Hazard ratio** | **p-value** | **Hazard ratio** | **p-value** |
| Systolic PA pressure (mmHg) | 1.02 [1.02; 1.03] | <0.001 | 1.01 [1.01; 1.02] | 0.002 | 1.03 [1.02; 1.03] | <0.001 |
| Diastolic PA pressure (mmHg) | 1.01 [1.00; 1.01] | <0.001 | 1.00 [1.00; 1.01] | 0.122 | 1.03 [1.02; 1.05] | <0.001 |
| Mean PA pressure (mmHg) | 1.03 [1.02; 1.03] | <0.001 | 1.01 [1.00; 1.03] | 0.029 | 1.03 [1.02; 1.04] | <0.001 |
| Mean RA pressure (mmHg) | 1.03 [1.02; 1.04] | <0.001 | 1.03 [1.01; 1.06] | 0.007 | 1.03 [1.02; 1.05] | <0.001 |
| Mean PAWP (mmHg) | 1.03 [1.01; 1.04] | <0.001 | 1.01 [0.99; 1.02] | 0.517 | 1.04 [1.02; 1.06] | <0.001 |
| SVO_2_ (%) | 0.96 [0.95; 0.97] | <0.001 | 0.98 [0.96; 0.99] | 0.001 | 0.95 [0.93; 0.96] | <0.001 |
| Pulmonary vascular resistance ([dyn*s/cm^5^) | 1.0014 [1.009; 1.019] | <0.001 | 1.0018 [1.0008; 1.0028] | <0.001 | 1.0011 [1.0004; 1.0018] | 0.001 |

PA = pulmonary artery, RA = Right atrial, PVR= pulmonary vascular resistance, PAWP = pulmonary capillary wedge pressure, SvO2= mixed venous oxygen saturation. Hazard ratios and p-values are the results of multiple Cox proportional hazards model. Each variable was entered separately into a model adjusted for age, creatinine, hs-cTnT, NTproBNP, sex, heart rate and presence of atrial fibrillation with the combined endpoint of all-cause mortality, heart transplantation or left ventricular assist device implantation as the dependent variable. Presence of ICM was further added in the overall cohort as an additional independent variable.

**Supplementary Table 8 -– Multiple Cox-Regression for the combined Endpoint – adjusted for history of atrial fibrillation**

|  | **Overall cohort**  **(n=784)** | | **Ischemic Cardiomyopathy (n=320)** | | **Non-ischemic cardiomyopathy (n=464)** | |
| --- | --- | --- | --- | --- | --- | --- |
| **Variable** | **Hazard ratio** | **p-value** | **Hazard ratio** | **p-value** | **Hazard ratio** | **p-value** |
| Systolic PA pressure (mmHg) | 1.02 [1.02; 1.03] | <0.001 | 1.01 [1.01; 1.02] | 0.002 | 1.03 [1.02; 1.03] | <0.001 |
| Diastolic PA pressure (mmHg) | 1.01 [1.00; 1.01] | <0.001 | 1.00 [1.00; 1.01] | 0.122 | 1.03 [1.02; 1.05] | <0.001 |
| Mean PA pressure (mmHg) | 1.03 [1.02; 1.03] | <0.001 | 1.01 [1.00; 1.03] | 0.029 | 1.03 [1.02; 1.04] | <0.001 |
| Mean RA pressure (mmHg) | 1.03 [1.02; 1.04] | <0.001 | 1.03 [1.01; 1.06] | 0.007 | 1.03 [1.02; 1.05] | <0.001 |
| Mean PAWP (mmHg) | 1.03 [1.01; 1.04] | <0.001 | 1.01 [0.99; 1.02] | 0.517 | 1.04 [1.02; 1.06] | <0.001 |
| SVO_2_ (%) | 0.96 [0.95; 0.97] | <0.001 | 0.98 [0.96; 0.99] | 0.001 | 0.95 [0.93; 0.96] | <0.001 |
| Pulmonary vascular resistance ([dyn*s/cm^5^) | 1.0014 [1.009; 1.019] | <0.001 | 1.0018 [1.0008; 1.0028] | <0.001 | 1.0011 [1.0004; 1.0018] | 0.001 |

PA = pulmonary artery, RA = Right atrial, PVR= pulmonary vascular resistance, PAWP = pulmonary capillary wedge pressure, SvO2= mixed venous oxygen saturation. Hazard ratios and p-values are the results of multiple Cox proportional hazards model. Each variable was entered separately into a model adjusted for age, creatinine, hs-cTnT, NTproBNP, sex and history of atrial fibrillation with the combined endpoint of all-cause mortality, heart transplantation or left ventricular assist device implantation as the dependent variable. Presence of ICM was further added in the overall cohort as an additional independent variable.
